# Phytochemical Phenolic Profile, Cholinesterase and α-Glucosidase Inhibitory Activities Rationalized by Molecular Docking, and Antioxidant Capacity of *Hibiscus sabdariffa*

**DOI:** 10.3390/molecules31193374

**Published:** 2026-09-23

**Authors:** Ashwell Rungano Ndhlala, Emrah Dikici, Sevgi Altın, Arzu Kavaz Yüksel, Mesut Işık, Ahmet Gokhan Aggul, Ekrem Köksal

**Affiliations:** 1 Green Biotechnologies Research Centre, School of Agricultural and Environmental Sciences, University of Limpopo, Private Bag X1106, Sovenga 0727, South Africa; ashwell.ndhlala@ul.ac.za; 2 Dogubayazit Ahmed-i Hani Vocational School, Agri Ibrahim Cecen University, Agri 04400, Türkiye; edikici@agri.edu.tr; 3 Central Researching Laboratory, Agri Ibrahim Cecen University, Agri 04100, Türkiye; 4 Department of Chemistry, Faculty of Science and Art, Erzincan Binali Yıldırım University, Erzincan 24100, Türkiye; sevgialtin2424@gmail.com (S.A.); ekoksal@erzincan.edu.tr (E.K.); 5 Department of Food Technology, Technical Sciences Vocational School, Atatürk University, Erzurum 25030, Türkiye; arzu-kavaz23@hotmail.com; 6 Department of Bioengineering, Faculty of Engineering, Bilecik Şeyh Edebali University, Bilecik 11230, Türkiye; 7 Department of Biochemistry, Faculty of Pharmacy, Agrı Ibrahim Cecen University, Agri 04100, Türkiye; agaggul@agri.edu.tr

**Keywords:** *Hibiscus sabdariffa*, phytochemical analysis, cholinesterase, glucosidase, LC-MS/MS, antioxidant activity

## Abstract

This study aimed to evaluate the antioxidant, anticholinesterase, and α-glucosidase (AG) inhibitory activities of the ethanolic leaf extract, characterize its phenolic composition, and investigate the interactions of the major identified phytochemicals with the target enzymes through molecular docking analysis. The phenolic profile was determined by LC–MS/MS, antioxidant activity was assessed using DPPH and ABTS radical scavenging assays, enzyme inhibitory activities against acetylcholinesterase (AChE), butyrylcholinesterase (BChE), and AG were evaluated in vitro, and molecular docking was performed to examine ligand–enzyme interactions. Among the analyzed compounds, quercetin, myricetin, fumaric acid, vanillic acid, 4-hydroxybenzoic acid, and salicylic acid were identified as the predominant phenolics in the leaf extract. The extract exhibited DPPH and ABTS radical scavenging activities of approximately 19% and 11%, respectively. It also demonstrated potent enzyme inhibitory activity, with IC_50_ values of 5.76 ± 0.21 µg/mL for AChE, 3.82 ± 0.17 µg/mL for BChE, and 6.24 ± 0.50 µg/mL for AG. Molecular docking analysis revealed that myricetin and quercetin exhibited the most favorable binding affinities toward AChE, BChE, and AG. Myricetin showed docking scores of −11.188, −9.642, and −7.899 kcal/mol against AChE, BChE, and AG, respectively, supported by hydrogen-bonding, π–π stacking, and hydrophobic interactions within the enzyme active sites. Re-docking validation yielded RMSD values below 2.0 Å for all targets, confirming the reliability of the docking protocol. These findings demonstrate that *H. sabdariffa* leaf extract is a rich source of bioactive phenolics with promising antioxidant and enzyme inhibitory properties, while molecular docking provides structural evidence supporting the observed biological activities.

## 1. Introduction

*Hibiscus sabdariffa* L. (HS), commonly known as roselle, is a medicinal and edible species in the family *Malvaceae* that is widely cultivated throughout tropical and subtropical regions for its nutritional, pharmaceutical, and industrial value [1,2]. The plant is recognized as a rich source of bioactive phytochemicals, including phenolic acids, flavonoids, anthocyanins, and organic acids, which contribute to its diverse biological properties [3,4,5,6]. Owing to this rich phytochemical composition, *H. sabdariffa* has attracted considerable attention as a natural source of antioxidant and health-promoting compounds.

Although the majority of phytochemical and pharmacological studies have focused on the calyces because of their commercial importance, the leaves also represent a valuable edible plant material that is traditionally consumed as leafy vegetables and incorporated into various culinary preparations in several African and Asian countries [6,7]. Recent investigations have shown that *H. sabdariffa* leaves are rich in phenolic compounds and flavonoids and possess antioxidant, antihyperlipidemic, antiatherosclerotic, antimicrobial, and antitumor activities [8,9,10,11,12,13,14,15,16,17,18,19]. Nevertheless, compared with the calyces, the chemical composition and biological potential of the leaves remain insufficiently characterized, highlighting the need for further phytochemical and pharmacological investigations.

Oxidative stress is closely associated with the pathogenesis of numerous chronic disorders, including Alzheimer’s disease (AD) and diabetes mellitus (DM) [20,21,22,23]. Consequently, naturally occurring phenolic compounds have received increasing attention because of their antioxidant capacity and their ability to modulate disease-related enzymes. AChE and BChE are established therapeutic targets for the symptomatic treatment of AD, as inhibition of these enzymes enhances cholinergic neurotransmission by increasing acetylcholine availability [24]. Likewise, inhibition of AG delays carbohydrate digestion and glucose absorption, thereby contributing to improved postprandial glycemic control in patients with DM [25]. Therefore, plant-derived phenolics capable of simultaneously inhibiting cholinesterases and AG may represent promising multifunctional agents for the management of neurodegenerative and metabolic disorders.

Molecular docking has become a valuable complementary tool for interpreting enzyme inhibitory activity by predicting the binding orientation and interactions of bioactive compounds within enzyme active sites. When integrated with in vitro biological assays, docking analysis provides molecular-level evidence supporting the observed inhibitory effects and facilitates the identification of compounds that may be primarily responsible for the biological activity.

The present study was undertaken to investigate the biological potential of the ethanolic extract obtained from *Hibiscus sabdariffa* leaves through the evaluation of its antioxidant, AChE, BChE, and AG inhibitory activities. To support these investigations, the phenolic profile of the extract was characterized by LC–MS/MS, and molecular docking analyses were performed to elucidate the interactions of the major identified phenolic constituents with the target enzymes, thereby providing a molecular basis for the experimentally observed enzyme inhibitory activities.

## 2. Results and Discussion

### 2.1. LC–MS/MS Phytochemical Characterization and Biological Activities of Hibiscus sabdariffa Extract

The LC-MS/MS analysis of the ethanol extract of HS demonstrated that the extract possesses a multicomponent phytochemical structure with a pronounced chemical richness in terms of flavonoids and phenolic acids. The detection of 18 out of the 20 targeted standard compounds (Table 1), with quercetin and myricetin as the predominant constituents and fumaric acid and vanillic acid among the major compounds that followed them, revealed that the extract exhibited a flavonol-dominant yet phenolic acid-rich profile.

Recent studies on roselle have likewise reported that HS extracts are rich in flavonoids, phenolics, tannins, and related secondary metabolites, and that although the chemical composition may vary depending on the extraction approach and the target analyte panel, the species generally possesses a phytochemical reservoir with high biological potential [26,27].

The identification of quercetin and myricetin as the two most dominant compounds in the present study is consistent with the flavonoid-centered chemical character previously reported for HS in the literature. Indeed, in the study by Choi et al. [27] compounds such as quercetin, luteolin, myricetin, and (+)-catechin were highlighted in *Hibiscus calyx* extract, and these compounds were emphasized as functional metabolites capable of interacting particularly with oxidative stress-related targets. Similarly, LC-MS analysis of roselle extracts indicated that the flavonoid and phenolic contents constitute the basis of the plant’s anti-inflammatory, antioxidant, and health-promoting properties [26]. Therefore, the high levels of quercetin and myricetin detected here suggest that a flavonol-dominant chemical architecture may underlie the biological effects of the extract.

From the perspective of the phenolic acid fraction, the remarkable levels of vanillic acid, fumaric acid, ellagic acid, caffeic acid, 4-hydroxybenzoic acid, and salicylic acid indicate that the extract possesses a diverse phenolic acid profile in addition to its flavonoid richness. LC–MS/MS-based studies conducted on different phenolic-rich plant matrices have demonstrated that secondary metabolite classes such as phenolic acids and flavonoids are associated with antioxidant and enzyme-modulating activities [25,28]. These studies provide a broader phytochemical context for interpreting the functional relevance of the corresponding metabolite classes identified in the present extract, rather than implying direct botanical or compositional equivalence between the investigated species. Accordingly, the high levels of vanillic acid and fumaric acid, together with the considerable levels of ellagic acid and caffeic acid, further demonstrate the chemical diversity of the H. sabdariffa leaf extract.

This chemical pattern also appears meaningful when compared with other plants belonging to the Malvaceae family. In an LC-MS/MS analysis of the ethanol extract of Abelmoschus esculentus flower, phenolic compounds were reported to be associated with antioxidant and anticholinergic potential, and the phenolic profile was particularly described as constituting the chemical basis of biological activity [29]. This similarity suggests that HS may likewise possess a functional repertoire of secondary metabolites rich in flavonoids and phenolic acids within the same family. Moreover, the profile identified here is also important in showing that roselle is not solely anthocyanin-based, but also exhibits strong chemical diversity in terms of flavonols and phenolic acids beyond anthocyanins. In particular, the co-occurrence of compounds such as quercetin, myricetin, kaempferol, and caffeic acid in the ethanol extract indicates that the phenolic character of the species should be evaluated within a broader framework [27,30].

The biological significance of the phenolic profile is particularly noteworthy in relation to pathophysiological processes based on oxidative stress and inflammation. It has been reported that HS can reduce malondialdehyde levels in cerebral ischemia–reperfusion injury, increase antioxidant defense parameters such as glutathione, superoxide dismutase, and catalase, and suppress inflammatory cytokines [31]. In addition, the ethanol extract of *Hibiscus calyces* has been shown to suppress fluoranthene-induced oxidative damage and ferroptosis in human keratinocytes, regulate AhR-related responses, and support antioxidant mechanisms [32]. When these studies are considered together, it may be suggested that the redox-active phenolics identified at high levels in the present study, such as quercetin, myricetin, ellagic acid, caffeic acid, and vanillic acid, could provide a chemical basis for the capacity of roselle extract to regulate oxidative stress responses.

The phenolic composition is also thought to have indirect importance in relation to neurodegenerative processes. In particular, the contribution of flavonoids and phenolic acids to the suppression of oxidative stress, inflammatory responses, and cellular damage in multifactorial neurodegenerative disorders such as Alzheimer’s disease has been widely discussed. More broadly, studies of phenolic-rich plant extracts have shown that secondary metabolite profiles comprising flavonoids and phenolic acids may be associated with antioxidant and enzyme-modulating activities relevant to neurodegenerative and metabolic targets [25,28,30,33,34]. In the present study, these reports provide a general structure–activity context for interpreting the potential biological relevance of the secondary metabolite classes identified in *H. sabdariffa*, rather than serving as direct interspecies comparisons. Although not directly studied in HS, the finding that the bud water extract of *H. syriacus*, which belongs to the *Hibiscus* genus, exhibits anti-obesity effects associated with metabolic disorders provides additional evidence suggesting the pharmacological importance of this genus in terms of bioactive metabolites [35]. Therefore, the flavonol- and phenolic acid-dominant profile identified here provides a meaningful chemical framework for multitarget herbal approaches that may gain importance in neurodegenerative and metabolic contexts.

Although compounds such as resveratrol, oleuropein, and phloridzin dihydrate were detected in low concentrations in the extract and were not quantitatively dominant, they still contribute to the preservation of chemical diversity. LC–MS/MS studies of phenolic-rich plant extracts have similarly demonstrated that plant matrices contain complex combinations of major and minor secondary metabolites associated with antioxidant and enzyme-modulating properties [25,28,33,36]. However, the present study does not allow the individual or combined contributions of these minor constituents to be established experimentally. Therefore, the chemical value of the HS extract should be evaluated not only in terms of high-level metabolites such as quercetin and myricetin, but also together with the complementary phenolic spectrum formed by lower-level compounds such as gallic acid, protocatechuic acid, luteolin, resveratrol, and phloridzin dihydrate. Furthermore, recent agricultural/ecophysiological studies on HS have shown that the species displays a biology sensitive to early developmental stages and environmental interactions, suggesting that phenolic composition may be influenced not only by the analytical method but also by cultivation and growth conditions. This latter inference should be regarded as a cautious evaluation based not on direct phenolic quantification but rather on the biological plasticity of the species [37].

In conclusion, the LC-MS/MS profile of the ethanol extract of HS revealed a phytochemical structure rich in flavonols and phenolic acids, with significant levels of compounds such as quercetin and myricetin, together with vanillic acid, fumaric acid, ellagic acid, caffeic acid, 4-hydroxybenzoic acid, and salicylic acid. This phenolic distribution appears to be consistent with the current literature regarding the antioxidant, anti-inflammatory, and health-promoting potential of roselle extracts [26,27,31,32].

The antioxidant activity of the ethanol extract of HS (Roselle) was evaluated in terms of its DPPH and ABTS radical scavenging potential, as well as its inhibitory effects on AChE, BChE, and AG enzymes. The inhibition curves are shown in Figure 1, and the calculated percentage inhibition and IC_50_ values are summarized in Table 2.

The DPPH radical scavenging activity of the HS extract was measured as 19.41%, while the ABTS radical scavenging activity was determined as 10.59%. These values are significantly lower compared to the standard antioxidant Trolox (DPPH: 74.53%, ABTS: 78.92). According to the results, the HS extract exhibited notable inhibitory activity against all tested enzymes. The IC_50_ value for AChE was 5.76 ± 0.21 µg/mL (R^2^ = 0.997), while for BChE it was 3.82 ± 0.17 µg/mL (R^2^ = 0.983). In comparison, the standard inhibitor tacrine showed stronger inhibition with IC_50_ values of 3.89 ± 0.28 µg/mL (R^2^ = 0.998) for AChE and 3.36 ± 0.32 µg/mL (R^2^ = 0.959) for BChE.

For AG inhibition, the *H. sabdariffa* leaf ethanolic extract exhibited an IC_50_ value of 6.24 ± 0.50 µg/mL, whereas the reference inhibitor acarbose showed a lower IC_50_ value of 4.00 ± 0.35 µg/mL, indicating stronger inhibitory potency. Although the reference inhibitors displayed lower IC_50_ values, as expected, the *H. sabdariffa* extract demonstrated considerable inhibitory activity within the same concentration range. Overall, these findings indicate that the leaf ethanolic extract possesses notable in vitro inhibitory activity against cholinesterases and AG.

Accumulating evidence suggests that Alzheimer’s disease and diabetes mellitus share several pathological mechanisms, including oxidative stress, chronic inflammation, insulin resistance, and impaired glucose metabolism [38]. Consequently, AChE, BChE, and AG have emerged as important molecular targets for the discovery of multifunctional bioactive compounds. In the present study, the low IC_50_ values obtained for all three enzymes indicate that the *H. sabdariffa* leaf extract contains constituents capable of inhibiting these enzymes in vitro. However, these findings should be interpreted as evidence of enzyme inhibitory potential rather than direct therapeutic efficacy.

LC–MS/MS analysis demonstrated that the extract is rich in phenolic acids and flavonoids, including quercetin, myricetin, fumaric acid, vanillic acid, ellagic acid, 4-hydroxybenzoic acid, salicylic acid, caffeic acid, kaempferol, butein, and protocatechuic acid. Many of these compounds have previously been reported to possess antioxidant, anti-inflammatory, neuroprotective, and enzyme inhibitory activities [29,30,39,40,41,42,43,44,45,46]. Therefore, the observed enzyme inhibitory effects may, at least in part, be attributed to the phenolic constituents identified in the extract.

The cholinesterase inhibitory activity of *H. sabdariffa* has been reported previously. Oboh et al. [47] demonstrated that phenolic extracts obtained from the calyces inhibited AChE (EC_50_ = 46.96 µg/mL) and BChE (EC_50_ = 40.38 µg/mL) in rat brain tissue in vitro. Similarly, Adewole et al. [48] reported that flavonoids identified in *H. sabdariffa*, including delphinidin-3-sambubioside, kaempferol-3-O-rutinoside, and quercetin-3-rutinoside, exhibited favorable binding affinities toward AChE, BChE, and monoamine oxidase-B, while molecular dynamics simulations supported the stability of the ligand–enzyme complexes. Yagi et al. [49] further reported that red calyx extracts exhibited higher biological activity than white varieties.

El-Shiekh et al. [50] found that both white and red hibiscus extracts showed moderate AChE inhibitory activity, with IC_50_ values of 123.22 ± 9.63 and 106.09 ± 7.17 µg/mL, respectively, although their activities were lower than that of galantamine. The same study also reported beneficial effects in an in vivo model, including attenuation of neuroinflammatory and amyloidogenic markers following administration of anthocyanin-rich hibiscus extracts. Although these findings support the pharmacological potential of *H. sabdariffa*, the present study evaluated only in vitro enzyme inhibition and therefore does not permit conclusions regarding in vivo neuroprotective efficacy.

The antidiabetic potential of *H. sabdariffa* has also been investigated in previous studies. Alegbe et al. [51] reported that aqueous calyx extracts inhibited both α-amylase and AG and reduced blood glucose levels in experimental animals. Likewise, Rasheed et al. [52] and Zulfiqar et al. [53] demonstrated dose-dependent AG inhibitory activity using extracts prepared by different extraction methods. Furthermore, Bule et al. [54] observed improvements in fasting plasma glucose and LDL cholesterol following *H. sabdariffa* consumption, while molecular modeling studies indicated that quercetin, hibiscetin, gossypetin, and protocatechuic acid exhibited favorable binding affinities toward phosphoenolpyruvate carboxykinase (PEPCK), a key enzyme involved in gluconeogenesis [55]. Although these findings suggest that *H. sabdariffa* may influence multiple metabolic targets, the present study specifically evaluated in vitro AG inhibition.

Taken together, the present results demonstrate that the ethanolic extract of *H. sabdariffa* leaves is a rich source of phenolic compounds exhibiting antioxidant activity together with significant in vitro inhibitory effects against AChE, BChE, and AG. The molecular docking analysis further supports the possible contribution of the major identified phenolic constituents to these enzyme inhibitory activities. While these findings highlight the potential of *H. sabdariffa* leaves as a promising source of bioactive compounds, additional in vivo investigations and clinical studies are required to confirm their biological efficacy and therapeutic relevance.

### 2.2. Molecular Docking Studies and Validation

The rational design of effective therapeutics depends on identifying molecules that engage specific biological targets with high selectivity and favorable pharmacological outcomes. Among the computational strategies developed to support this process, molecular docking has emerged as a particularly valuable tool, allowing researchers to predict how ligands orient and bind within a target protein’s active site. By estimating the energetic favorability and structural stability of the resulting complexes, docking provides an efficient means of prioritizing candidate compounds before experimental validation. In line with this approach, molecular docking simulations were performed to predict the inhibitory potential of the most abundant natural compounds identified in the *H. sabdariffa* extract against the target enzymes, based on their predicted binding affinities and interaction profiles. The reliability of the docking protocol was further confirmed through re-docking validation, and the resulting binding scores and interaction patterns are presented in Table 3.

Evaluation of the docking scores and Glide Emodel values revealed that all the compounds exhibited inhibitory effects against AChE, BChE, and AG enzymes. Among these compounds, myricetin and quercetin displayed the most favorable inhibitory profiles against all three target enzymes. Since these two compounds yielded closely comparable docking scores, Emodel values, which are used to rank ligand binding poses based on the overall protein–ligand interaction energy, were additionally considered to further differentiate their relative binding efficiencies, and myricetin emerged as the most effective compound among the tested compounds.

Detailed analysis of the binding mode of myricetin, one of the major phytochemical constituents identified in the extract, demonstrated a favorable interaction profile within the active site of AChE (Figure 2). In terms of binding affinity, myricetin yielded a docking score of −11.188 kcal/mol, which was more favorable than that of the reference inhibitor tacrine (−9.301 kcal/mol), suggesting a stronger predicted interaction with the catalytic binding site of AChE. Myricetin formed hydrogen bond interactions with Phe295 through its carbonyl and hydroxyl groups, while an additional hydrogen bond was established with Ser293 via one of its hydroxyl groups. Moreover, the aromatic scaffold of myricetin exhibited a π–π stacking interaction with Tyr124, contributing to the stabilization of the ligand within the catalytic gorge. The ligand was further surrounded by several hydrophobic residues, including Trp286, Val294, Phe297, Phe338, Tyr337, and Tyr341, which enhanced the stability of the ligand–enzyme complex through hydrophobic contacts. Collectively, these hydrogen-bonding, π–π stacking, and hydrophobic interactions provide a structural explanation for the strong binding affinity of myricetin toward AChE and are consistent with its experimentally observed inhibitory activity.

The binding mode of myricetin within the active site of BChE revealed a network of favorable interactions that contributed to its high binding affinity (Figure 3). In terms of binding affinity, myricetin yielded a docking score of −9.642 kcal/mol against BChE, which was more favorable than that of the reference inhibitor tacrine (−8.360 kcal/mol), suggesting a stronger predicted interaction with the active site of the enzyme. Myricetin established hydrogen bond interactions with Ala328, Glu197, Gly115, and Trp82 through its hydroxyl groups, thereby stabilizing the ligand within the binding pocket. In addition, the ligand was surrounded by several hydrophobic residues, including Phe329, Tyr332, Met434, Met437, Tyr440, Ile442, Trp430, Tyr128, Leu125, and Tyr114, which further enhanced the stability of the ligand–enzyme complex through hydrophobic contacts. These interactions collectively explain the favorable docking score of myricetin and provide structural evidence supporting its potent inhibitory activity against BChE.

The binding mode of myricetin within the active site of AG revealed a favorable interaction pattern that contributed to its strong binding affinity (Figure 4). Against AG, both myricetin and quercetin exhibited comparable binding affinities, with docking scores of −7.899 and −8.299 kcal/mol, respectively. Although quercetin showed a slightly more favorable docking score toward AG, the difference between the two compounds was relatively small. Considering the overall docking performance across all three target enzymes (AChE, BChE, and AG), together with the Glide Emodel values, myricetin displayed a more consistent and favorable binding profile. Therefore, myricetin was selected as the representative compound for detailed interaction analysis. Myricetin formed hydrogen bond interactions with Asp327 (protonated; ASH327), Asp443, and Tyr605 through its hydroxyl groups, contributing to the stabilization of the ligand within the binding pocket. In addition, a π–π stacking interaction was observed with Phe575, further strengthening ligand binding. The ligand was also surrounded by several hydrophobic residues, including Tyr299, Ile328, Trp406, Ile364, Trp441, Met444, and Trp539, which further stabilized the ligand–enzyme complex through hydrophobic contacts. Collectively, these hydrogen-bonding, π–π stacking, and hydrophobic interactions provide a structural basis for the favorable binding affinity of myricetin toward AG and support its potent inhibitory activity.

To validate the reliability of the docking protocol, the co-crystallized ligands were re-docked into the active sites of AChE, BChE, and AG. The superposition of the experimentally determined and re-docked ligand conformations yielded RMSD values of 0.1866 Å, 1.6459 Å, and 1.6160 Å, respectively (Figure 5), indicating that the docking protocol reliably reproduced the experimentally observed binding poses.

## 3. Materials and Methods

### 3.1. Chemicals, Reagents, and Instruments

All chemicals, reagents, and enzymes used in this study were of analytical grade. AG (EC 3.2.1.20) from *Saccharomyces cerevisiae* (Type I, C5003), AChE (EC 3.1.1.7) from Electrophorus electricus (Type VI-S, C3389), and BChE (EC 3.1.1.8) from equine serum (C7512) were obtained from Sigma-Aldrich (St. Louis, MO, USA). Tacrine and acarbose were used as positive controls in the cholinesterase and AG inhibition assays, respectively. All chemicals and solvents were of analytical grade and were purchased from commercial suppliers.

The qualitative and quantitative characterization of phenolic compounds was performed using liquid chromatography coupled with tandem mass spectrometry (LC–MS/MS). Absorbance measurements for the antioxidant and enzyme inhibition assays were carried out using a double-beam UV–Vis spectrophotometer (SQ4802, UNICO, Dayton, NJ, USA).

### 3.2. Plant Material and Extract Preparation

The red leaves of *H. sabdariffa* L. were collected from Antalya Province, Türkiye, in May 2025. The collection region is geographically located between 36–37° N latitude and 27–34° E longitude. Taxonomic identification and authentication of the plant material were performed at the Department of Biology, Erzincan Binali Yıldırım University, Türkiye, and the species was confirmed as *H. sabdariffa* L. The collected leaves were dried in an incubator at 40 °C for 72 h and subsequently ground into a fine and homogeneous powder using a mechanical grinder. The powdered plant material was transferred into airtight sample containers and stored at 4 °C until extraction.

The ethanolic extract was prepared according to the procedure described by Altay et al. [56]. Briefly, 20.0 g of dried and powdered *H. sabdariffa* leaf material was extracted with 400 mL of a 70% ethanol–water mixture (*v*/*v*), corresponding to a plant material-to-solvent ratio of 1:20 (*w*/*v*). The extraction was performed by stirred maceration in a sealed vessel under continuous magnetic stirring at ambient laboratory temperature for 24 h. Following extraction, the mixture was filtered through Whatman No. 1 filter paper (Sigma-Aldrich, Darmstadt, Germany) to remove the solid plant residues. The resulting filtrate was concentrated under reduced pressure using a rotary evaporator (Heidolph 94200, Bioblock Scientific, Burladingen, Germany) until the solvent was completely removed. The concentrated extract was subsequently dried under cold-air conditions to obtain a dry extract and stored at −20 °C until further analysis. The extraction process yielded approximately 20–25% (*w*/*w*) dry extract. All extraction and analytical procedures were performed in triplicate to ensure reproducibility.

### 3.3. Phenolic Compound Analysis by LC-MS/MS

The qualitative and quantitative profiles of phenolic compounds present in the ethanol extract of HS were determined using liquid chromatography–tandem mass spectrometry (LC-MS/MS; Shimadzu, Kyoto, Japan). Before analysis, the method was optimized using 20 high-purity standard compounds. Based on the signal responses of the standards, the system conditions were optimized, and subsequently, the quantitative determination of the relevant compounds in the *H. sabdariffa* ethanol extract was carried out. The methodological framework was adapted based on the approach reported by Altay et al. [56], while Yılmaz’s [57], comprehensive LC-MS/MS validation study was consulted in terms of selectivity and quantitative reliability.

Chromatographic separation was performed on a C18 Inertsil ODS-4 column (3.0 × 100 mm, 2 µm particle size). The column temperature was maintained at 40 °C throughout the analysis, the injection volume was set at 5 µL, and the mobile phase flow rate was set at 0.5 mL/min. The mobile phase system consisted of deionized water containing 0.1% formic acid as solvent A and methanol containing 0.1% formic acid as solvent B. The separation was carried out using a gradient elution program; initially, the system was operated with a composition of 80% A and 20% B, reaching 50% A/50% B at the 8th minute, shifting to 5% A/95% B at the 12th minute, and returning to the initial composition at the 15th minute for column re-equilibration. The total analysis time, including the final equilibration step, was 15 min. This gradient program enabled the efficient separation of phenolic compounds with different polarities within a single analytical run.

The analyses were carried out using modules compatible with the Shimadzu liquid chromatography system, including a column oven (CTO-10ASVP), degasser unit (DGU-20A3R), dual pump system (LC-30AD), and autosampler (SIL-30AC). This modular configuration contributed to analytical performance by ensuring flow stability, removing air bubbles from the mobile phase, enabling reproducible sample injections, and maintaining constant column conditions. Thus, the separation of target phenolic compounds in the *H. sabdariffa* ethanol extract was conducted under controlled and reproducible chromatographic conditions.

Mass spectrometric determinations were performed in Multiple Reaction Monitoring (MRM) mode using the LabSolutions software (Ver. 5.128). In MRM mode, the mass spectrometer selectively monitors the molecular ions of the target analytes along with their characteristic product ions, thereby recording only the signals from the relevant compounds. This targeted scanning approach improves the sensitivity and reliability of the method by reducing matrix-related interferences and background noise commonly encountered in plant extracts. Therefore, all quantitative calculations in this study were performed based on MRM measurements. In particular, this approach provided an important analytical advantage for the selective determination of phenolic acids, flavonoids, and other phytochemicals present at low levels in the same sample.

Identification of the target analytes was based on two complementary criteria: (i) agreement between the retention time of each sample peak and that of the corresponding authentic standard analyzed under identical chromatographic conditions and (ii) confirmation of the compound-specific precursor-to-product ion transition in the appropriate electrospray ionization mode. The ESI modes, MRM transitions, LOD and LOQ values, calibration equations, and coefficients of determination for the 20 reference standards are provided in Table S1. The R^2^ values ranged from 0.994 to 1.000, indicating satisfactory linearity for the target analytes. Because the analyses were conducted in targeted MRM mode, compound-specific tandem mass spectrometric information was recorded and reported as characteristic precursor-to-product ion transitions, together with the corresponding ESI modes, in Table S1. Analytes producing signals below their corresponding LOD values were reported as not detected (ND), whereas analytes producing signals between the LOD and LOQ were classified as detected but not quantified (DNQ). Only analytes producing signals above their corresponding LOQ values were quantified using the respective calibration equations. Recovery values were expressed as percentages and calculated as the ratio of the measured concentration to the corresponding spiked concentration multiplied by 100. The concentrations reported in Table 1 refer to the final LC–MS/MS analytical solution prepared from the ethanolic extract and do not represent dry-weight-normalized concentrations of the original plant material. A representative LC–MS/MS chromatogram of the standard phenolic compound mixture used for the identification and quantification of the target analytes is provided in Figure S1.

### 3.4. Radical Scavenging Assays (DPPH and ABTS Methods)

The antioxidant capacity of HS extract was comprehensively evaluated using DPPH and ABTS assays, which are radical scavenging methods. For the DPPH assay, a fresh 0.26 mM ethanolic solution of 2,2-diphenyl-1-picrylhydrazine was prepared. The ethanol extract was tested to determine its free radical scavenging activity at various stock concentrations. The sample was mixed with an equal volume of the DPPH solution and incubated in the dark at room temperature for 30 min [43,58]. To account for any interference arising from the intrinsic color of the extract, appropriate sample blanks containing the extract without DPPH were prepared and used for blank correction. Following incubation, the decrease in absorbance was measured at 517 nm using a UV–Vis spectrophotometer. Radical scavenging activity was calculated as the percentage of inhibition, and IC_50_ values were obtained from the dose–response curve.

In parallel, the ABTS radical cation was generated by reacting ABTS with potassium persulfate and allowing the mixture to stand in the dark at room temperature for approximately 14 h to ensure complete oxidation. Before analysis, the ABTS•^+^ solution was diluted with phosphate buffer or deionized water to achieve an absorbance of 0.750 ± 0.025 at 734 nm. Subsequently, different concentrations of the extract were added to the prepared radical solution, and the decrease in absorbance at 734 nm was measured spectrophotometrically [59]. Scavenging efficiency was expressed as IC_50_ values, defined as the concentration required to neutralize 50% of the ABTS radicals.

### 3.5. Anticholinesterase Inhibition

The spectrophotometric approach described by Ellman et al. [60] was used to evaluate the inhibitory effect of the plant ethanol extract on AChE and BChE enzymes. The reaction mixture was prepared using Tris-HCl buffer (pH 8.0), cholinesterase enzyme solution, 5,5′-dithio-bis(2-nitrobenzoic) acid (DTNB), and a suitable substrate. Acetylthiocholine iodide was used as the substrate for AChE, and butyrylthiocholine iodide for BChE. The extract whose inhibitory potential was to be determined was added to the reaction medium in different doses. To ensure enzymatic hydrolysis, the reaction mixture was incubated at 25–30 °C. Absorbance measurement was performed at a wavelength of 412 nm. Tacrine was used as a positive control. Activity (%)-concentration graphs were plotted to determine the concentrations (IC_50_) that inhibited 50% of AChE and BChE enzyme activities [33,61]. To ensure the accuracy and reproducibility of the findings, each concentration was tested in three experiments.

### 3.6. AG Inhibition

The inhibitory effect of the plant extract on AG was determined according to the method proposed by Tao et al. [62]. The reaction mixture consisted of an AG enzyme solution, extract samples prepared at various concentrations, and p-NPG substrate mixed in phosphate buffer (pH 6.8). To ensure enzymatic hydrolysis, the processes were incubated at an appropriate temperature. The absorbance of p-nitrophenol, formed by the degradation of the substrate by enzymes, was read at 405 nm. Acarbose was used as the standard substance. The IC_50_ value of the extract was determined using dose–response curves [25,61]. Each experiment was repeated three times to verify the accuracy of the findings.

### 3.7. Molecular Docking Analysis

Molecular docking studies were performed to evaluate the binding interactions of the most abundant natural compounds identified in *H. sabdariffa* extract with AChE (PDB: 4M0E), BChE (PDB: 5NN0), and AG (PDB: 3L4Y), using Maestro 12.9 (Schrödinger Suite, Schrödinger LLC, New York, NY, USA). Protein structures, retrieved from the RCSB Protein Data Bank, were prepared using the Protein Preparation Wizard (water removal, hydrogen addition, and energy minimization with the OPLS force field), and receptor grids were generated around the co-crystallized ligands to define the active sites. Ligand structures were built in Maestro and optimized with LigPrep (2026-2), then docked into the respective active sites using Glide XP. Tacrine and acarbose, obtained from PubChem, served as reference inhibitors and were processed under the same protocol. Binding affinities were evaluated based on GlideScore and Emodel values, and the lowest-energy poses were analyzed for key interactions (hydrogen bonding, hydrophobic contacts, π–π stacking). Docking protocol validity was confirmed by re-docking co-crystallized ligands, with RMSD values < 2 Å indicating reliable predictions [63,64].

### 3.8. Statistical Analysis

All experiments were carried out in triplicate to ensure consistency and reproducibility. Results are expressed as mean ± standard deviation (SD). Statistical analyses were performed using one-way analysis of variance (ANOVA) in GraphPad Prism (version 8). When significant differences were detected, multiple comparisons among groups were performed using Tukey’s post hoc test. Different uppercase letters indicate statistically significant differences at *p* < 0.05.

## 4. Conclusions

The findings of this study demonstrate that HS leaves, which are traditionally consumed as a leafy vegetable in many regions, represent a valuable source of polyphenolic compounds, predominantly chlorogenic acid and its isomeric derivatives, together with quercetin- and kaempferol-based glycosides. The abundance of these phytochemicals is likely to contribute to the antioxidant and anti-inflammatory potential of the leaves [65]. Collectively, these findings provide scientific support for the traditional consumption of HS leaves as a functional food and suggest their potential application as a natural source of bioactive ingredients for functional beverages and other health-promoting food products [66]. Consumption of HS, which is abundant in anthocyanins and other bioactive phytochemicals, significantly reduced postprandial glycaemic and insulinaemic responses. Although inhibition of AG activity is considered a plausible mechanism underlying these effects, the observed responses are likely multifactorial and may also be influenced by physicochemical characteristics of the intervention, including its acidic pH.

## Figures and Tables

**Figure 1:**
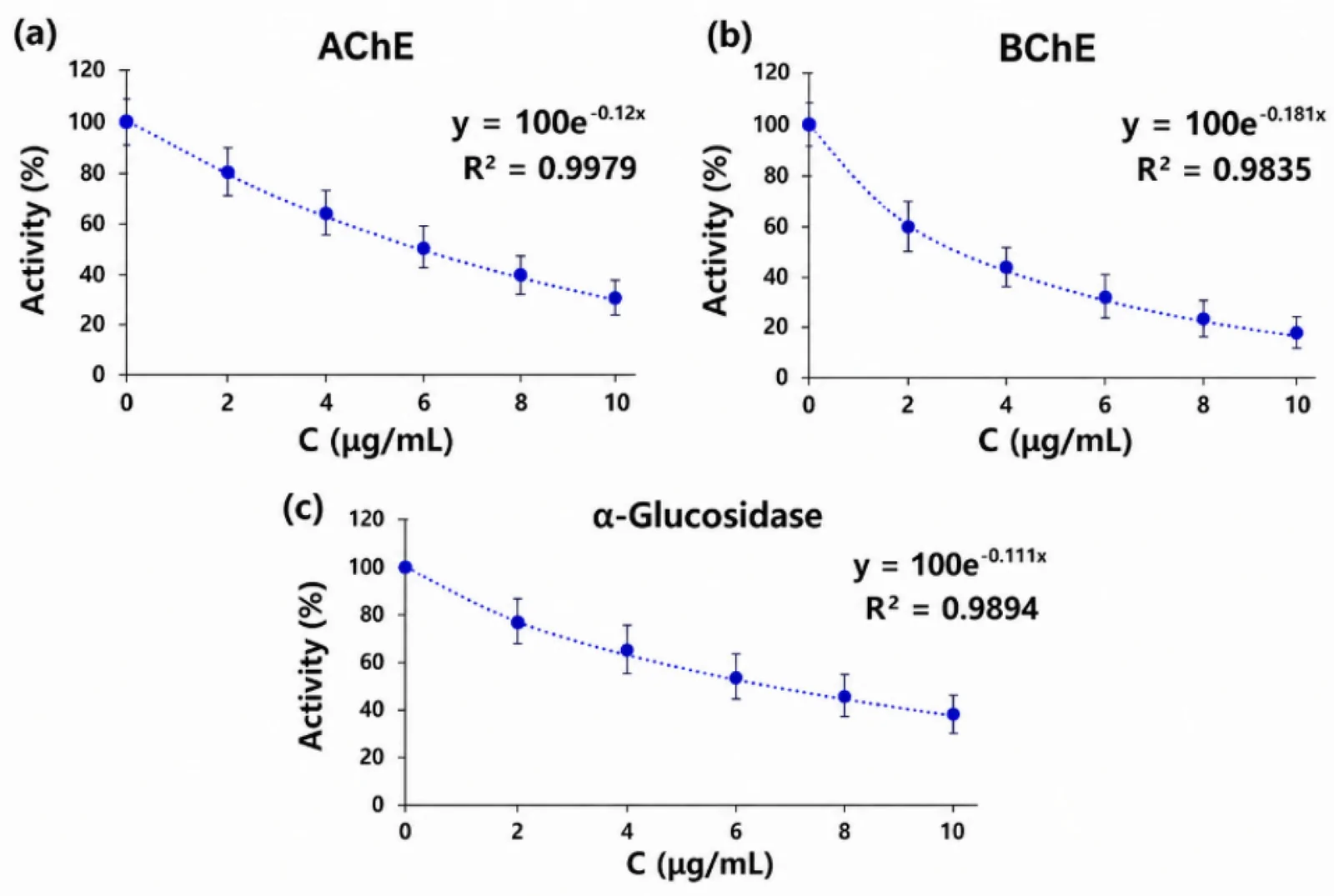
Concentration–response curves of the inhibitory effects of *H. sabdariffa* leaf ethanolic extract against (**a**) AChE, (**b**) BChE, and (**c**) AG.

**Figure 2:**
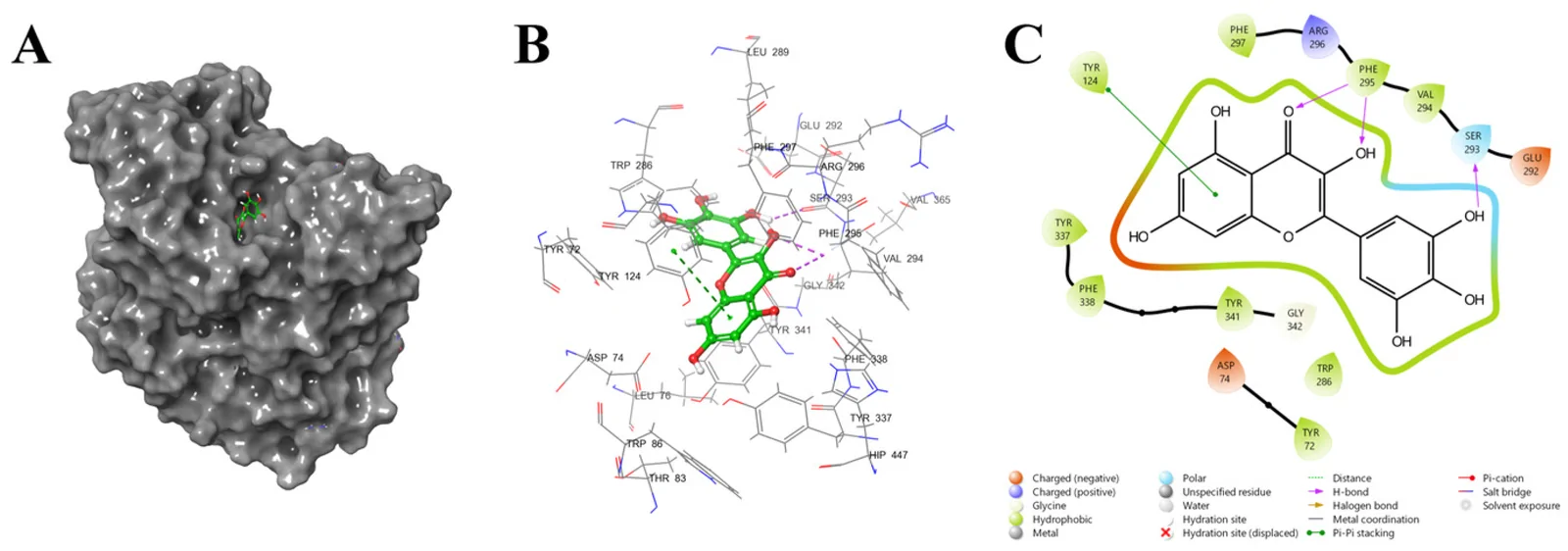
Structural representations of the interactions between myricetin and AChE (4M0E). For the myricetin, the three-dimensional structure of the complex (**A**), the binding site view (**B**), and the two-dimensional interaction diagram (**C**) are shown. Myricetin is depicted in green.

**Figure 3:**
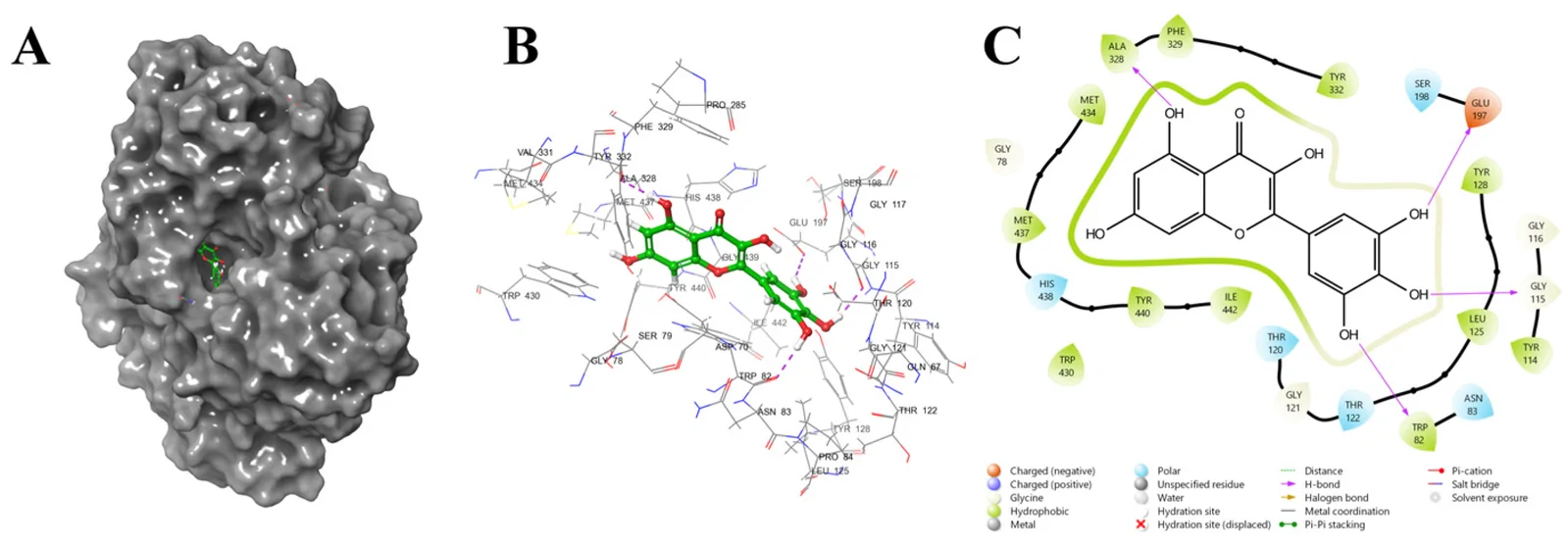
Structural representations of the interactions between myricetin and BChE (5NN0). For the myricetin, the three-dimensional structure of the complex (**A**), the binding site view (**B**), and the two-dimensional interaction diagram (**C**) are shown. Myricetin is depicted in green.

**Figure 4:**
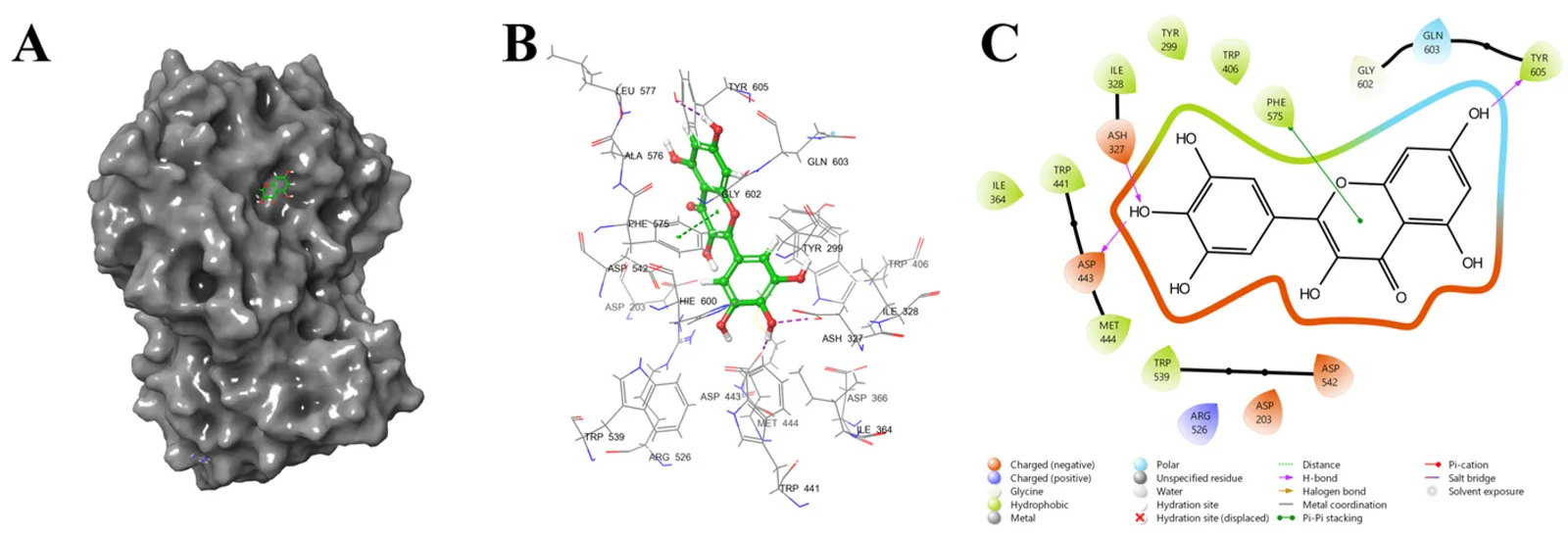
Structural representations of the interactions between myricetin and AG (3L4Y). For the myricetin, the three-dimensional structure of the complex (**A**), the binding site view (**B**), and the two-dimensional interaction diagram (**C**) are shown. Myricetin is depicted in green.

**Figure 5:**
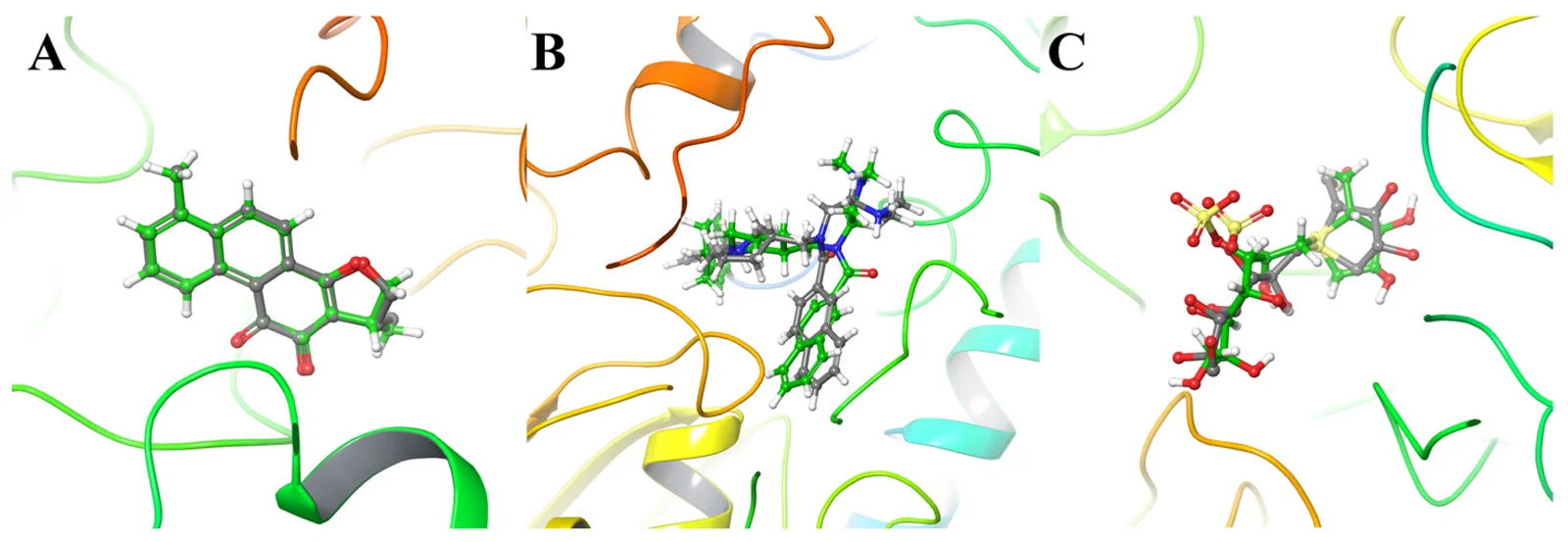
Re-docking validation of AChE (**A**), BChE (**B**), and AG (**C**) enzymes for the co-crystallized ligands 1YL, 92H, and NR4, respectively. The co-crystallized ligands are shown in grey using stick representation, while the docked ligands are depicted in green using the same representation.

**Table 1:** Phenolic content of *H. sabdariffa* ethanol extract.

Compounds	^a^ LOD/LOQ (μg/L)	Recovery (%)	^b^ RT (min.)	Conc. (µg/L)
Quercetin	22.5/25.7	100.1	6.091	4274.29
Acetohydroxamic Acid	2.8/8.2	100.0	1.986	46.35
Catechin hydrate	8.2/11.4	99.4	4.958	46.67
Vanillic Acid	125.5/142.2	100.1	6.026	1423.50
Resveratrol	9.0/13.6	99.8	5.713	9.84
Fumaric Acid	25.2/31.3	99.7	3.674	1638.02
Gallic acid	0.90/1.6	100.0	4.134	36.59
Caffeic Acid	6.3/10.7	100.9	5.283	329.19
Phloridzin dihydrate	61.0/207.0	100.0	5.646	DNQ
Oleuropein	0.05/1.0	99.7	5.643	41.26
Ellagic Acid	0.101/0.333	100.2	5.895	901.91
Myricetin	55.4/59.6	99.9	5.858	3634.59
Protocatechuic acid	30.3/35.4	101.1	5.875	109.61
Butein	22.7/28.6	99.6	6.084	118.55
Naringenin	5.4/6.4	99.8	6.104	N.D.
Luteolin	0.5/2.5	100.7	6.19	13.18
Kaempferol	206.6/214.3	99.9	6.288	273.71
Alizarin	65.2/77.5	96.6	6.8	N.D.
4-Hydroxybenzoic Acid	30.5/40.25	99.6	6.13	658.38
Salicylic acid	4.2/7.6	100.9	6.104	592.25

^a^ LOD/LOQ (µg/L): limit of detection/limit of quantitation, ^b^ RT: retention time. N.D: not detected. DNQ: detected but not quantified; the analyte signal was detected but was below the corresponding LOQ.

**Table 2:** Comparison of antioxidant and enzyme inhibitory activities of *H. sabdariffa* leaf extract and reference standards.

Inhibitor	DPPH ^a^ (0.2 μg/mL)	ABTS ^a^ (0.2 μg/mL)	AChE IC_50_^b^ (μg/mL)	BChE IC_50_^b^ (μg/mL)	AG IC_50_^b^ (μg/mL)
*H. sabdariffa*	19.41 ± 1.32	10.59 ± 0.83	5.76 ± 0.21 ^B^	3.82 ± 0.17 ^B^	6.24 ± 0.5 ^B^
Tacrine			3.89 ± 0.28 ^A^	3.36 ± 0.32 ^A^	-
Acarbose			-	-	4.00 ± 0.35 ^A^
Trolox	74.53 ± 4.95	78.92 ± 5.26			

^a^ Values are expressed as percent radical scavenging activity. ^b^ Reference control. IC_50_ values are expressed as mean ± SD (*n* = 3). Different uppercase letters within the same column indicate statistically significant differences (*p* < 0.05).

**Table 3:** Docking scores and binding energies of the bioactive compounds for the target enzymes.

	AChE (PDB Code: 4M0E)		BChE (PDB Code: 5NN0)		AG (PDB Code: 3L4Y)	
Compounds	Docking Score (kcal/mol)	Glide Emodel (kcal/mol)	Docking Score (kcal/mol)	Glide Emodel (kcal/mol)	Docking Score (kcal/mol)	Glide Emodel (kcal/mol)
Myricetin	−11.188	−56.467	−9.642	−61.178	−7.899	−56.605
Quercetin	−10.689	−54.652	−10.115	−55.285	−8.299	−53.398
Vanillic Acid	−5.415	−31.748	−4.494	−23.979	−5.639	−15.337
Fumaric Acid	−1.577	−7.209	−1.408	−12.045	−2.069	−12.387
Tacrine ^[a]^	−9.301	−45.507	−8.360	−47.535	-	-
Acarbose ^[b]^	**-**	**-**	-	**-**	−13.360	−73.404

^[a]^ Tacrine is a standard inhibitor of ChE enzymes. ^[b]^ Acarbose is a standard inhibitor of AG enzyme.

## Data Availability

The original contributions presented in this study are included in the article/Supplementary Materials. Further inquiries can be directed to the corresponding author.

## Supplementary Materials

The following supporting information can be downloaded at: https://www.mdpi.com/article/10.3390/molecules31193374/s1, Figure S1: Representative LC-MS/MS chromatogram for standard phenolic compounds. Table S1. LC–MS/MS analytical parameters of the standard compounds, including electrospray ionization modes, multiple reaction monitoring (MRM) transitions, limits of detection and quantification (LOD/LOQ), calibration equations, and coefficients of determination (R^2^).

## Abbreviations

The following abbreviations are used in this manuscript:

HS	*Hibiscus sabdariffa*
LC-MS/MS	Liquid Chromatography–Tandem Mass Spectrometry
DPPH	2,2-Diphenyl-1-picrylhydrazyl
FRAP	Ferric Reducing Antioxidant Power
ABTS	2,2′-Azino-bis(3-ethylbenzothiazoline-6-sulfonic acid)
AChE	Acetylcholinesterase
BChE	Butyrylcholinesterase
AG	α-Glucosidase
DTNB	5,5′-Dithiobis-(2-nitrobenzoic acid)
p-NPG	p-nitrophenyl-D-glucopyranoside
