# Supplementary material for: Phytochemical Phenolic Profile, Cholinesterase and α-Glucosidase Inhibitory Activities Rationalized by Molecular Docking, and Antioxidant Capacity of Hibiscus sabdariffa
Source: Molecules. 2026 Sep 23;31(19):3374. doi: 10.3390/molecules31193374 (PMC13666032; doi:10.3390/molecules31193374)
Supplement: Supplementary file 1 [file molecules-31-03374-s001.zip › Supplemantary materials_ TABLE S1.pdf]

Table S1. LC–MS/MS analytical parameters of the standard compounds, including electrospray ionization modes, multiple reaction monitoring (MRM) transitions, limits of detection and quantification (LOD/LOQ), calibration equations, and coefficients of determination (R^2).

Standard Compounds	ESI Mode	MRM Transition (m/z)	LOD/LOQ (µg L^{-1})	R^2	Equation
Quercetin	neg	301.1 > 151	22.5/25.7	0.999	Y = (13.7831)X + (−146.951)
Acetohydroxamic Acid	pos	76.10 > 43.10	2.8/8.2	0.999	Y = (150.982)X + (23.1833)
Catechin hydrate	neg	291.10 > 139.00	8.2/11.4	0.999	Y = (79.2933)X + (−2406.22)
Vanillic Acid	pos	168.80 > 93.00	125.5/142.2	0.998	Y = (48.0522)X + (−876.904)
Resveratrol	pos	229.10 > 135.00	9.0/13.6	0.998	Y = (46.4361)X + (−1314.61)
Fumaric Acid	neg	115.20 > 71.00	25.2/31.3	0.999	Y = (20.2986)X + (−762.592)
Gallic acid	neg	169.20 > 125.00	0.90/1.6	0.999	Y = (65.3835)X + (−2699.84)
Caffeic Acid	neg	179.20 > 135.00	6.3/10.7	0.996	Y = (124.785)X + (−487.132)
Phloridzin dihydrate	neg	435.00 > 273.10	61.0/207.0	0.999	Y = (33.4069)X + (−1396.90)
Oleuropein	neg	539.10 > 377.20	0.05/1.0	0.999	Y = (25.9240)X + (−558.916)
Ellagic Acid	neg	300.90 > 145.10	0.101/0.333	1.000	Y = (5.25903)X + (−1167.31)
Myricetin	neg	317.10 > 150.90	55.4/59.6	0.999	Y = (37.0934)X + (2684.23)
Protocatechuic acid	neg	181.20 > 108.00	30.3/35.4	0.994	Y = (526.954)X + (23026.1)
Butein	neg	271.10 > 135.00	22.7/28.6	0.999	Y = (49.3543)X + (367.917)
Naringenin	neg	271.10 > 150.90	5.4/6.4	0.996	Y = (317.241)X + (33733.3)
Luteolin	neg	285.20 > 132.90	0.5/2.5	0.998	Y = (34.6668)X + (3721.79)
Kaempferol	neg	285.10 > 116.90	206.6/214.3	0.999	Y = (2.63905)X + (−206.494)
Alizarin	neg	239.20 > 210.90	65.2/77.5	0.998	Y = (3.97487)X + (1614.23)
4-Hydroxybenzoic Acid	neg	137.20 > 93.00	30.5/40.25	0.999	Y = (735.804)X + (−498.102)
Salicylic acid	neg	137.20 > 93.00	4.2/7.6	0.999	Y = (746.369)X + (6072.41)

ESI, electrospray ionization; MRM, multiple reaction monitoring; LOD, limit of detection; LOQ, limit of quantification; R^2, coefficient of determination.
